# Advances in Management of Fournier’s Gangrene by Coupling Intensive Hospital Treatment With Innovative Post-discharge Hyperbaric Oxygen Therapy Rehabilitation: A Case Report

**DOI:** 10.7759/cureus.36639

**Published:** 2023-03-24

**Authors:** Barbara Wójcik, Jerzy Superata, Tomasz Tuleja, Ewa Kobielska, Zbigniew Szyguła

**Affiliations:** 1 Department of Clinical Rehabilitation, University of Physical Education, Krakow, POL; 2 Burns and Plastic Surgery Centre of Malopolska, Department of Adult Plastic and Reconstructive Surgery, Ludwik Rydygier Memorial Hospital, Krakow, POL; 3 Burns and Plastic Surgery Centre of Malopolska, Department of Hyperbaric Oxygen Therapy, Ludwik Rydygier Memorial Hospital, Krakow, POL; 4 Department of Sport Medicine and Human Nutrition, University of Physical Education, Krakow, POL

**Keywords:** fournier's gangrene, rehabilitation, necrotizing soft tissue infection (nsti), hyperbaric oxygen therapy (hbot), sepsis

## Abstract

Fournier's gangrene (FG) is a rare form of necrotizing soft tissue infection characterized by an acute, aggressive, and rapidly progressive course. In this case report, we describe advanced therapy combining critical care, surgery, pharmacotherapy, extended biochemical/cellular blood diagnostics, and post-discharge hyperbaric oxygen therapy rehabilitation. Such an intervention resulted in survival and improved health status and quality of life of the patient with FG and septic shock.

## Introduction

Fournier's gangrene (FG), a rare, life-threatening necrotizing infection affecting the perineum, perineal region, and genitals, is frequently misdiagnosed because of the nonspecific nature of its symptoms. In the absence of prompt and adequate treatment, FG can spread rapidly along the fascia plane, eventually leading to septic shock in a short time [1]. Despite advances made in comprehension of its pathophysiology, the associated mortality rate (from 20% to 88%) does not appear to have reduced over the past 25 years [2]. The complex and destructive nature of FG implicates multidisciplinary therapeutic approaches which evolve gradually. The timing, importance, extent, and details of the applied procedures are still a matter of controversy and discussion [3,4].

Moreover, the successful in-hospital stabilization of a patient’s condition does not guarantee full recovery. Even those who survive often necessitate multiple reconstructive surgeries, which may present a risk to immunocompromised sepsis survivors who are unable to accept skin grafts and suffer from poor wound healing [5].

Here, we present a case report of a patient who survived and finally recovered from FG and septic shock due to the timely and synergistic action of critical care support, surgery, antibiotics, blood purification, and bimodal (“acute” and “chronic”) hyperbaric oxygenation, supported by extended biochemical/cellular blood diagnostics.

## Case presentation

**Figure 1:**
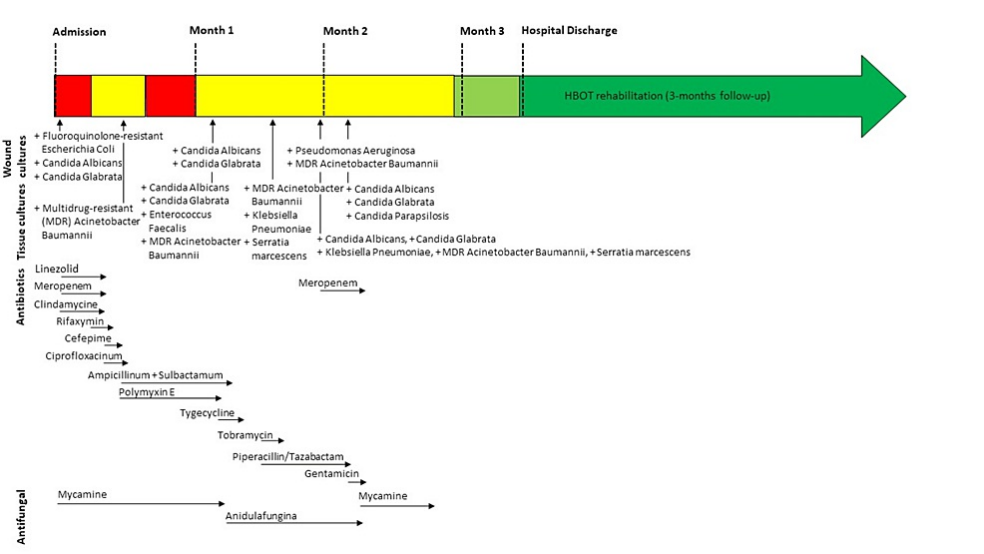
Time course of antimicrobial therapy throughout hospitalization demonstrating the positive tissue cultures and antimicrobial therapies.

A previously healthy 52-year-old man was admitted to the hospital in general critical condition after cardiac arrest and with rapidly progressing necrotizing soft tissue infection (NSTI) of his buttocks and perineum. On the same day, FG was diagnosed. The patient’s NSTI continued to advance and was accompanied by septic shock. Tissue cultures demonstrated a polymicrobial infection; therefore, treatment consisted of broad-spectrum antibiotics (BSAs) (Figure 1).

**Figure 2:**
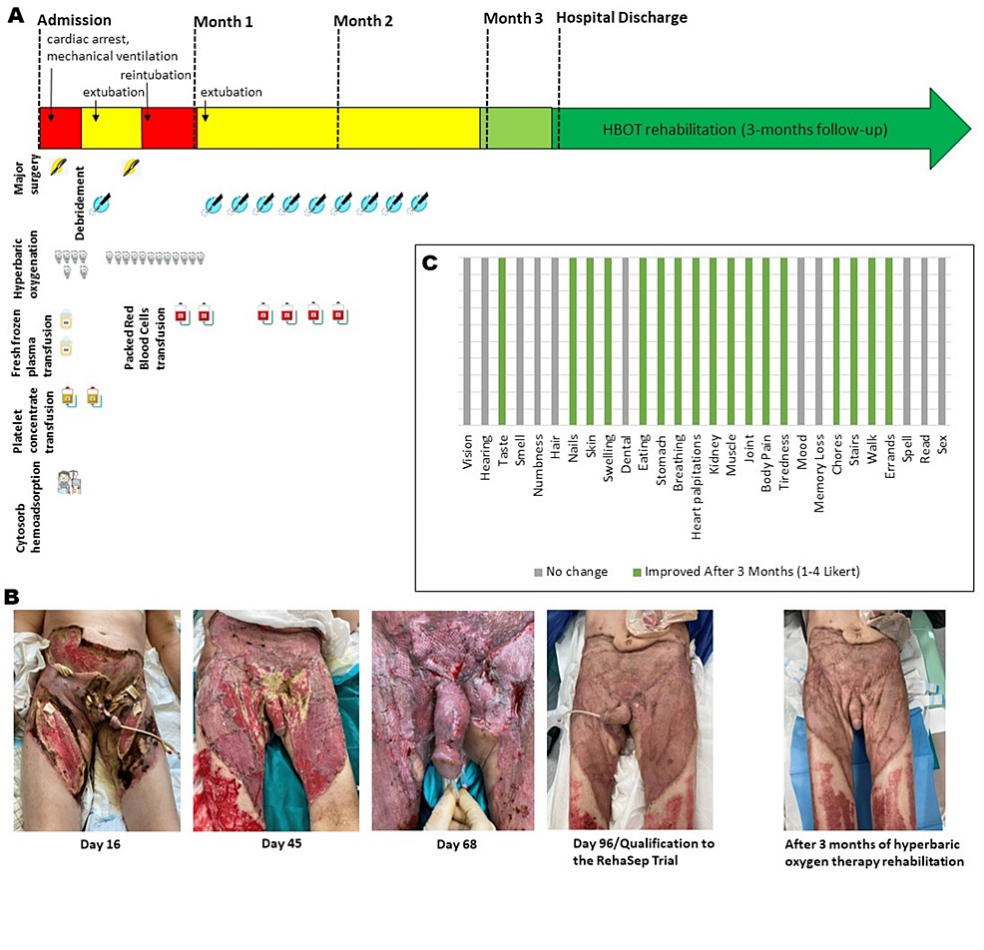
Time course of surgical therapy throughout hospitalization followed by three months of rehabilitation. This timeline demonstrates (A) occurrences of the major surgeries and surgical debridements, hyperbaric oxygen therapy sessions, CytoSorb hemoadsorption, transfusions (of fresh frozen plasma, platelet concentrate, packet red blood cells), and the associated changes in the patient’s clinical condition; (B) photographs illustrating the progression of the infection with necrotic regions, which resolved following multi-parameter diagnostically monitored therapy; and (C) changes in physiologic and physical functions after three-month hyperbaric oxygen therapy rehabilitation. Each green bar represents the improvement (according to a Likert score of 1-4) or no change after rehabilitation.

Fluid resuscitation, urine output monitoring, and four days of hemoadsorption using the extracorporeal adsorption device (CytoSorb, https://cytosorb-therapy.com/) were also implemented. Additionally, the patient underwent bilateral orchiectomy and colostomy, and he was aggressively treated with daily or alternating-day operative debridement of the necrotic tissues regarding the entire perineum, inguinal regions, bilateral thighs, buttocks, and circumferential abdominal wall. At the same time, he started emergency hyperbaric oxygen therapy (HBOT) sessions lasting 90 minutes at 2.5 atm - three sessions within 24 hours from admission, four sessions within 72 hours, and 11 subsequent sessions once a day. After seven days, the patient’s condition improved, and he was extubated. However, due to a high risk of fecal contamination, infected anal sphincter, and large perianal defects, a colostomy was performed. After that, the patient’s condition worsened, and he went into acute respiratory failure. X-ray examination revealed pulmonary lesions that were consistent with bilateral interstitial pneumonia. The patient was intubated again, and nebulized colistin therapy was administrated. After the next six days, the patient’s condition improved, and sedation was discontinued (Figure 2).

**Figure 3:**
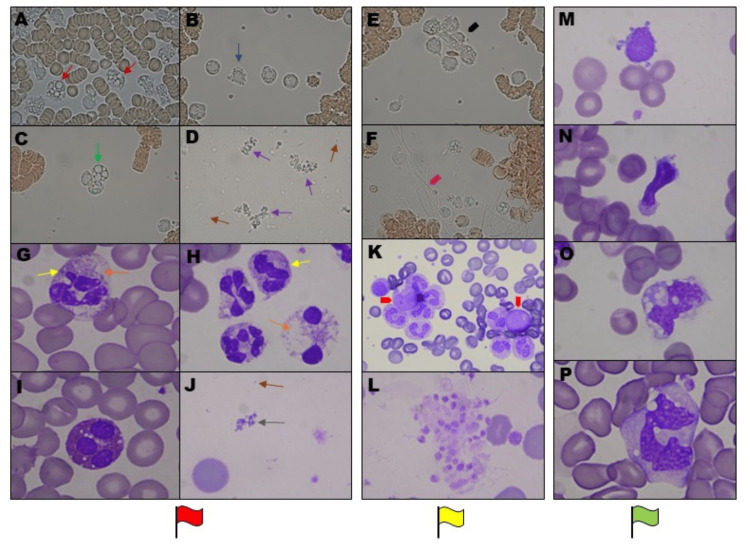
Characteristic patient blood cell morphological changes during the entire hospitalization and recovery period. Peripheral (heparinized) blood buffy-coat live-cell observation representative pictures showing (A) changes in neutrophils as ”toxic vacuolizations” with no ability of active movement (red arrows), (B) leukocyte apoptotic blebbing (blue arrow) as prominent surface protuberances and (C) exacerbated vacuolization of the cells (green arrow), (D) numerous microparticles (brown arrows) and bizarre free-floating structures (violet arrows) as the red flags (during patient’s critical condition), (E) leukocyte aggregates (black arrowhead), and (F) excessive platelet activation associated with fibrous deposits (pink arrowhead) as the yellow flags (during the serious but stable patient’s general condition), magnification of x400. Concomitant peripheral blood smear (EDTA) demonstrating: (G,H) apoptotic neutrophils with toxic granulations (yellow arrows), excessive vacuolization (orange arrows), (I) apoptotic and vacuolated eosinophils, (J) the dramatic number of microparticles (brown arrow) and free-floating structures (gray arrow) as the red flags, (K) immature leukocyte aggregates (green arrowheads) and (L) large platelet aggregates as the yellow flags, (M,N) growing number of atypical lymphocytes and (O,P) active monocytes as the green flags during patient’s condition improvement phase, magnification of x1000 and x400 in K.

From the day of patient admission, detailed microscopic examinations of blood smears and live-cell imaging of leukocyte activity were periodically performed during the entire hospitalization. The observations showed characteristic blood cell morphological changes, which correlated with the selected results of routine laboratory tests and the patient’s general condition. As the indications, we marked characteristic blood changes with red flags (during the patient’s critical state), yellow (as a reflection of the serious but stable general condition of the patient), and green flags (during the patient’s condition improvement phase) (Figure 3), (Table 1).

**Table 1:** Median and standard deviation (mean±STD) of selected blood test results which correlate with the characteristic blood cell morphological changes and patient’s general condition.

Test	Reference range	Red flags (patient’s critical state)	Yellow flags (serious but stable general condition of the patient)	Green flags (patient’s condition improvement phase)
White blood cell count	4-10 x 10^3^/μL	18.6±4.9	14.3±0.36	10.7±1.78
Neutrophils	1.6-7 x 10^3^/μl	12.52±0.9	8.7±0.8	4.43±0.3
Lymphocytes	0.8-4.5 x 10^3^/μl	1.42±0.26	2.63±0.5	3.78±0.31
Monocytes	0.2-1.2 x 10^3^/μl	0.59±0.06	0.65±0.02	1.67±0.2
Immature granulocyte count	0-0.03 x 10^3/μl	2.45±0.24	0.7±0.11	0.05±0.01
Erythrocytes	3.5-5.2 x 10^6/μL	2.6±0.15	3.44±0.09	4.31±0.25
Hemoglobin	11-15 g/dL	8.06±0.17	11.06±0.62	12.6±0.9
Hematocrit	34-45%	22.8±1.42	32.3±1.2	39.2±2.3
Platelets	150-400 x 10^3/μl	53.5±11.5	183.5±36.6	369.3±61.4
Fibrinogen	2-4 g/L	9.38±1.2	6.3±0.7	4.4±0.9
D-dimer	<500 ng/mL	13551±813.7	4748.8±674.7	1257.5±341.3
Procalcitonin	<0.5 ng/mL	70.4±33.4	1.93±0.06	0.09±0.04
C-reactive protein	<0.5 mg/L	367.2±75.4	110.4±25.3	27.2±21.4
Creatinine	58-96 μmol/L	39.5±3.2	50.3±2.5	68.5±4.03

Just after hospital discharge, the patient started the experimental three-month diagnostically monitored rehabilitation program in the form of modified HBOT (three times a week, 36 sessions of breathing 100% oxygen at 2.5 atm for 90 minutes). According to the RehaSep trial protocol, a wide range of physiological, hematological (Figure 3M-3P), and biochemical (blood tests) parameters were assessed at hospital discharge and during the subsequent three months in order to monitor changes in physical capacity, immunity, and degree of post-sepsis organ damage/recovery [6]. The patient, discharged immobile in a wheelchair, progressively regained walking ability within a three-month HBOT rehabilitation period. The total work ((kJ) measured by a cycloergometer) achieved by the patient in the ECG exercise test at the beginning and end of rehabilitation increased by 195% (from 20 to 59 kJ). In the quality of life assessment using the “Life after sepsis survey,” the patient reported that the majority of the assessed physiologic and functional capacities were significantly improved (Figure 2C) [7].

## Discussion

FG is a special form of NSTI characterized by an acute, aggressive, and rapidly progressive course [8]. During the development of NSTI, the microbial virulence caused by the toxins produced via the involved bacteria outweighs the host defense system, thus, promoting the rapid spreading of the infection and fulminant tissue destruction [9]. Concomitantly, this carries a substantial risk of the dysregulated host response to infection that can lead to sepsis and sepsis-associated multi-organ failure [10]. During sepsis, there is a full-blown, systemic activation of immune responses due to the massive release of pathogen- and damage-associated molecular patterns (PAMP, DAMP) from invading microorganisms and/or damaged host tissues, which leads to the overstimulation of immune cells, eventually resulting in immunosuppression. Moreover, sepsis is accompanied by a markedly imbalanced cytokine response (“cytokine storm”) that converts responses that are normally beneficial in the case of fighting infections into excessive, damaging inflammation [10]. Therefore, early resection of necrotic and infected tissues and appropriate BSA therapy are cornerstones of NSTI management [8]. Therefore, the immune system supported by BSAs has better odds of controlling the infection. In several studies, it was found that early surgical treatment reduces not only the mortality rate but also the risk of septic shock and the length of hospital stay [9,11-13]. However, there is still no consensus concerning a potential cut-off point for the “golden time-frame” in which the initial surgery should be performed [9].

It has been proved that the complete blood cell count (CBC) with differentials may contain a considerable amount of information, often providing early clues to the diagnosis of sepsis. Nonetheless, emerging evidence allows to suggest that emphasis should be shifted toward the neutrophil-to-lymphocyte ratio and, perhaps, the fraction of immature granulocytes emerging about one day after clinical infection [14]. Therefore, not only the CBC test and clinical condition of the patient but also microscopic observation of leukocyte activity as well as blood plasma features should determine the course of therapeutic actions.

Based on our observations, the increased number of blood microparticles is the main visible sign of the PAMP/DAMP release leading to a reduction in spontaneous leukocyte activity that accompanies the deterioration of a patient's condition. Our observations suggest that assessing the individual trajectory of the patient's leukocyte activation, functions, and interactions may prove crucial in deepening our understanding of this complex disease process, as immune cells are engaged from the very beginning to the very end - their composition, behavior, and secretions are indices of FG/sepsis dynamics. We are convinced that intensive efforts should be made to further develop cell analysis methods better applicable in medical diagnostics, providing the key to novel and effective therapies.

During sepsis, oxygen consumption at the cellular and mitochondrial levels is drastically impaired [15]. On the other hand, severe wounds and tissue damages are characteristic of FG and other NSTIs. Hence, HBOT with its immunostimulatory, angiogenic, and antimicrobial effects is now frequently used as adjunctive critical care treatment [16,17].

Yet, in this case (besides the critical care phase), we have applied HBOT as the rehabilitation treatment for an FG/sepsis survivor for the first time. Our experimental therapy was accompanied by extensive biochemical and microscopic monitoring to secure the patient’s safety. This attempt proved safe, successful, and measurably effective.

## Conclusions

NSTI with septic shock is a very serious and complex medical condition that should be under the care of an interdisciplinary team with access not only to the best surgical and critical care but also to sepsis experts who can help find the best way and time for appropriate treatment. Additionally, in contrast to the present fragmented primary care-based scheme, the continuity of integrated and coordinated care after hospital discharge is essential in the improvement of patient health and well-being, which defines a long-awaited successful management of FG.

HBOT could be recommended as a new opportunity for therapy continuation, especially among patients with contraindications to exercise training (e.g., unhealed wounds) within the key “therapeutic window” for rehabilitation just after hospital discharge. Nonetheless, in our opinion (based on our growing post-sepsis therapy experience), the HBOT rehabilitation sessions should be scheduled on alternate days in order to offset the transient effects of oxidative stress, thus, improving treatment tolerance and patient safety. In addition, considering the same number of sessions prescribed, the therapy is prolonged (in comparison to the conventional daily schedule), letting more time for wounds/transplants to heal completely.
